# Management of the Uncommon Bladder Cancers: A Single-Center Experience over 10 Years

**DOI:** 10.1155/2020/7563703

**Published:** 2020-10-07

**Authors:** Youssef Kadouri, Salim Lachkar, Hamza Dergamoun, Hachem El Sayegh, Lounis Benslimane, Yassine Nouini

**Affiliations:** Mohammed V University, Faculty of Medicine and Pharmacy of Rabat, Ibn Sina Hospital, Department of Urology A, Rabat, Morocco

## Abstract

**Background:**

Under the name of uncommon bladder cancers are gathered rare histological entities which represent less than 5% of bladder tumors. There is not a clear and consensual therapeutic management for these entities.

**Purpose:**

To review a single-institution 10-year experience with rare form of bladder cancers detailing the diagnosis, treatment, and patient outcome.

**Materials and Methods:**

We performed a retrospective review of 27 medical records of rare bladder cancer form treated at our center between February 2006 and February 2015. The clinicopathologic features are reported with emphasis on treatment and survival.

**Results:**

Mean patient age was 65.5 ± 20 yr and 70% of patients were males. Smoking background was found in 16 cases, chronic bladder irritation factors were found in 12 cases, and past urinary tract infection was found in 11 cases. The main symptom was total hematuria (93%) causing an anemia in 16 cases. The two mean histological forms were epidermoid carcinoma (37%) and adenocarcinoma (22%). 26% of patients were found to have extended invasive tumors (T4) at diagnosis. Metastatic disease was confirmed in 8 cases. Our patients were managed by a wide range of therapeutic modalities as total cystectomy with bilateral lymph node dissection (63%), palliative chemotherapy (30%), or concomitant radiochemotherapy (7%). 55.6% of patients were alive one year after diagnosis. Epidermoid carcinoma has the best prognosis followed by leiomyosarcoma and sarcomatoid carcinoma. Neuroendocrine carcinoma has the worst outcome. The overall 5-year survival rate is 33.3%.

**Conclusion:**

The rarity and small size of these tumors justify the absence of clear and consensual therapeutic management. No role of total cystectomy concerning the conclusions could be drawn but elements suggest this may be the treatment of choice. The highly aggressive nature of those lesions justifies an aggressive and fast therapy when feasible which gives the best outcomes.

## 1. Introduction

Bladder cancer is the 2nd urological cancer by frequency with 2.7 million people diagnosed worldwide each year and an incidence constantly increasing by about 1% per year [1, 2]. 95% of histological types are urothelial carcinoma. Rare form of bladder tumor does exist, including heterogeneous histological groups. They can constitute the main part of the tumor process or coexist with other more frequent components. There is not a specific genetic predisposition. Rare bladder tumors have some main risk factors: tobacco, exposition to aniline or alkylating agent (as cyclophosphamide), and pelvic irradiation. Their relative rarity provides difficulty to gain experience in the management of these tumors and could justify the lack of a clear and consensual therapeutic management. A review of our center experience over a 10-year period was undertaken to try to define more clearly their clinical features and response to treatment.

## 2. Materials and Methods

We performed a retrospective review of 27 medical records of rare bladder cancer form treated at our center, Urology « A » department of Ibn Sina Hospital in Rabat, between February 2006 and February 2015. The clinicopathologic features are reported with emphasis on treatment and survival.

The data collected for carrying out this work comes from patient files in the department's archives and operating reports.

After histological confirmation, 27 out of a total of 574 patients with bladder tumors were seen to have rare bladder carcinoma, an incidence of 4.1%. Under the “rare bladder carcinoma” term, all histological forms that are not urothelial carcinoma were included.

Patient age, sex, medical background, clinical history, physical examination data, histology, radiology and biology, treatment technique, and outcomes data were collected and summarized here.

The discussion is about a review of the literature, using the PubMed database and guidelines from urological and oncological learned societies. We detail many aspects of the diagnosis and treatment of bladder's rare tumors and we emphasize their prognostic factors and evolution.

## 3. Results

Over the data collecting period, there were 574 cases of bladder cancer. 27 were not urothelial carcinoma, with an incidence of 4.1%.

### 3.1. Epidemiology

There were 19 men (70%) and 8 women for an average age of 65.5 ± 20 years. Men were diagnosed earlier than women, respectively, at 60 and 71 years of age.

Regarding the medical background, 16 cases (59.3%) of smoking habits were found, whether active (14 cases) or passive (2 cases). The average consumption was 27 pack-year and 7 patients were not weaned at the diagnosis.

### 3.2. Medical Background

Chronic irritation factors were found in 12 cases (44%), as urolithiasis (6 cases), perineostomy due to a urethra trauma (2 cases), a four-time endoscopic urethrotomy due to a postinfectious urethra stenosis (1 case), urinary incontinence (2 cases), and a treated bilharzia infection (1 case). Bacteria's urinary tract infections were found in 11 cases (40.7%).

### 3.3. Presenting Symptoms

The presenting symptoms in the group are summarized in Table 1. Total hematuria was the main symptom, found in 25 cases (93%). Hematuria was painless in 14 patients (51.9%), with the remaining 11 (40.7%) having associated symptoms of bladder irritation (frequency, urgency, nocturia, or dysuria). The pathology was also revealed by an acute urinary retention (1 case) or a hypogastric budding lesions (1 case).

### 3.4. Physical Examination

The results of physical examination are grouped in Tables 1 and 2. On general physical examination, vitals were stable in all cases. 21 patients (77%) had abnormality as a hypogastric mass (4 cases), a mattery hypogastric fistula (2 cases), a bladder globe (4 cases), a hypogastric sensitivity (3 cases), a previous laparotomy scar (1 case), and groans on auscultation (3 cases).

More specifically, the urogenital and pelvic examination showed an enlarged prostate (7 cases), a pelvic shield (1 case), and an anterior vaginal wall's infiltration (4 cases), that is to say, 50% of the female patients.

### 3.5. Biological and Bacteriological Findings

The complete blood counts were often abnormal mainly due to anemia (16 cases, 60%), with a hemoglobin level ranging from 6.7 g/dl to 11.2 g/dl., caused either by the neoplastic disease or hematuria spoliation. Blood transfusion was required in 11 cases, therefore 62% of anemic patients. There were 6 cases of renal acute failure, twice treated by percutaneous nephrostomy. Significant bacteriuria (greater than 10^5^ organisms per ml) was identified at presentation in 18 (66.7%) of 27 patients where the midstream specimen of urine MSU results was available.

### 3.6. Radiology and Endoscopic Findings

**Figure 1:**
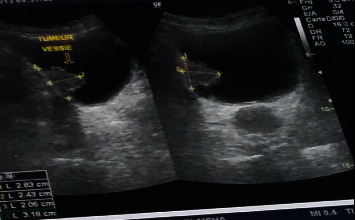
Bladder ultrasound showing a hyperechoic image of the right lateral wall.

24 patients had an abdominal ultrasonography examination (Figure 1). Ultrasonography of abdomen revealed a parietal tissue mass in 23 cases (85.2%) and a pyelocaliceal cavities dilation (10 cases), completed 1 time by an intravenous urography showing a lacunar image amputating right bladder horn and vesical dome with left ureterohydronephrosis.

A unique transurethral resection of the bladder (TURB) was performed for all patients. TURB was incomplete in 18 cases (66.7%) due to an endoscopically uncontrollable tumor. The diagnosis was made at endoscopy in all cases. Tumors were recorded as solitary in 21 patients and multiple in 6. The sites occupied by tumor were very variable (Table 3) but there appeared to be a slight predilection for the trigonal area and lateral walls. Tumors were usually described as solid, necrotic, or sessile and were frequently extensive, in 20 patients occupying 2 or more of the bladder areas listed in Table 3.

### 3.7. Anatomopathology

**Figure 2:**
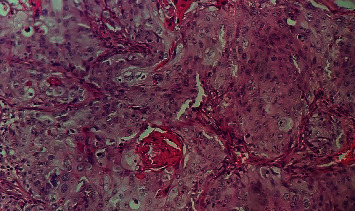
Moderately differentiated and keratinizing squamous cell carcinoma of the bladder mucosa (HEx100).

**Figure 3:**
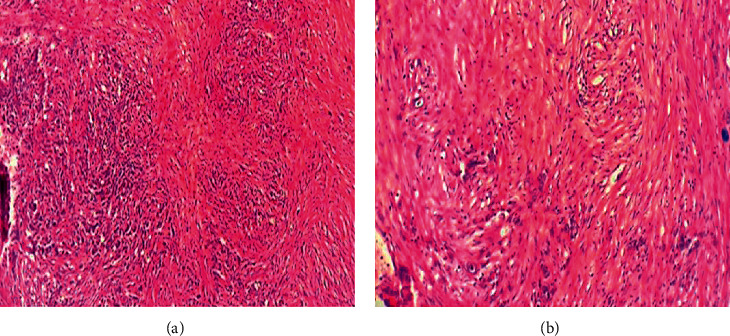
Sarcomatoid carcinoma presenting round cells, giant pleomorphic cells, and spindle-shaped anisokaryocytic cells. (A) HE original magnification ×100. (B) HE original magnification ×200.

**Figure 4:**
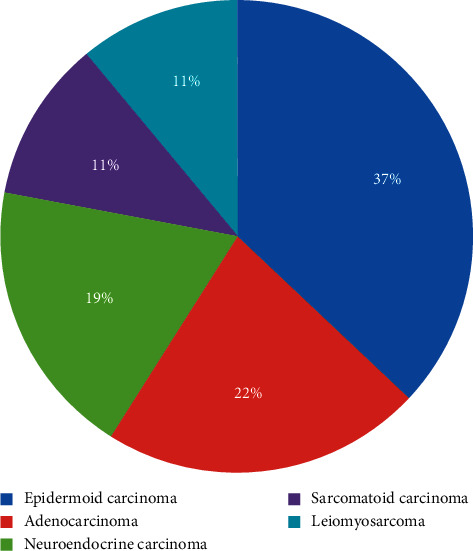
Distribution of cases collected in the department according to the histological type.

The anatomopathological examination of resection shavings is the key for the diagnosis (Figures 2 and 3).  Figure 4 shows the histological results. The two mean histological forms were the epidermoid carcinoma (37%) and the adenocarcinoma (22%), followed by neuroendocrine carcinoma (19%), sarcomatoid carcinoma (11%), and leiomyosarcoma (11%).

### 3.8. Tumor Grading

**Figure 5:**
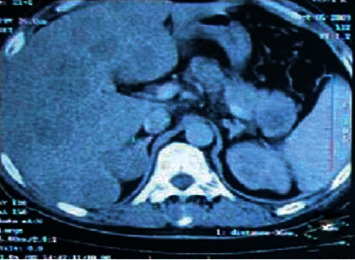
Abdominal CT showing diffuse secondary hepatic locations.

The tumor extension staging is based on thoracoabdominopelvic scanner. The CT scan was performed for all patients and revealed a perivesical fat tumor extension in 21 cases (78%). 78% of patients were found to have extended invasive tumors (T4) at diagnosis. The others found were an iliac lymphadenopathy (10 cases, 37%), intraparenchymal pulmonary nodules considered as pulmonary metastasis (6 cases), a case of rectosigmoid junction tumoral invasion, and a case of hepatic and retroperitoneal nodules (Figure 5). Bones scintigraphy was performed 3 times without revealed abnormality. Metastatic disease was confirmed in 8 cases.  Table 4 summarizes the tumor's grading.

### 3.9. Treatment and Survival

Our patients were managed by a wide range of therapeutic modalities. Total cystectomy associated with a bilateral ilioobturator lymph node dissection was performed for 17 patients (63%) whether male treated by total cystoprostatectomy (11 cases) or female by anterior pelvectomy (6 cases). Urinary diversion consisted in a wide range of techniques: enterocystoplasty (1 case) for a T2N0M0 young male, Bricker construction (9 cases), and cutaneous ureterostomy (7 cases) for altered general condition or patients appearing severely malnourished. Sometimes radical surgery was completed by adjuvant chemotherapy (5 cases). Two patients (7%) were treated with concomitant radiochemotherapy after refusing surgery.

As indicated 8 times (30%), palliative chemotherapy was not performed twice either for patient's refusal or for altered general condition.

Immediate postoperative complications were a day 6 abdominal collection, a hemorrhagic shock requiring intensive care (2 cases), and a case of day 7 death following respiratory distress and ischemic stroke.

The average long-term follow-up was 5 years. Favorable evolution occurred in 16 cases (60%). 55.55% of patients were alive one year after diagnosis. There were 4 patients with an unfavorable becoming: metastatic relapse at 5 months with hepatic, right adrenal and retroperitoneal metastases (1 case), and death at month 5 due to palliative treatment refusal (1 case) and death after the start of the chemotherapy (2 cases) at day 14 and month 4.

Survival at 1 and 5 years was heterogeneous depending on the tumor histological type. The epidermoid carcinoma has the best prognosis with a 70% 1-year survival and a 50% at 5-year survival. Leiomyosarcoma and sarcomatoid carcinoma has the second best outcomes with same results: 66.6% at 1 year and 33.3% at 5 years. Neuroendocrine carcinoma has the worst outcome with a 0% of 5-year survival. The overall survival rate of our series is 55.6% at 1 year and 33.3% at 5 years. Six patients were lost to follow-up.

Table 5 summarizes the survival depending on tumor histological type.

## 4. Discussion

Uncommon bladder tumors are very heterogeneous histological entities. Their aggressive nature and poor prognosis are a challenge for the medical team. However, because of their low incidence, there is not a clear consensual therapeutic management.

### 4.1. Vesical Epidermoid Carcinoma

It is the second more frequent type after urothelial carcinoma, with an overall average frequency of 2 to 5%. Epidermoid carcinomas are more common in the Middle East countries, Southeast Asia, and South America where its frequency can reach 20 to 30% of all bladder tumors. Its event is the first histological subtype in Egypt, before urothelial carcinoma. A possible explanation is the endemic bilharzia state in those countries. Links between bilharzia and epidermoid carcinoma have been widely proved, especially by Mostofi et al. with the chronic bladder epithelium irritation theory [3]. Other risk factors are smoking, neurological bladders, radiotherapy, chronic urinary tract infections, and intravesical foreign bodies. In our studies, 70% of patients had a chronic bladder irritation.

Hematuria is the main clinical sign. Other possible symptoms are voiding disorders as pollakiuria, voiding burns, pelvic pain, and low back pain related to tumor extension. Bladder irritants leading to tumor carcinogenesis are themselves sources of previous clinical sign. A physician has to avoid being falsely reassured by a too obvious clinical sign explanation that could hide a beginning tumor process.

Cystoscopy generally finds a compact nodule or plaque tumor. Bilharzia origin has a pearly coating and non-bilharzia forms an ulcerating appearance. Lesion is most often unique. Some diffuse cases have been reported.

Diagnosis is based on anatomopathological bladder tissue examination obtained by TURB. Epidermoid nature is confirmed by finding keratinizing differentiation and/or intercellular bridges. A transitional cell contingent can coexist, leading to the urothelial carcinoma with epidermoid inflection diagnosis.

Radical cystectomy with pelvic lymph node dissection and urinary diversion is the main treatment for such tumor type. Béjany and al. observed 40% urethral recurrences after cystectomy. Their team recommends a systematic urethrectomy [4]. Concomitant radiochemotherapy is an alternative for no-resectable tumor or surgery refusal. Immunotherapy appears to be a future possible promising approach as complement to surgery.

### 4.2. Primary Vesical Adenocarcinoma (PVA)

This is the third histological type with a 2% average frequency.

PVA generally appears between 60 and 70 years [5, 6]. The etiopathogenesis remains hypothetical. Adenocarcinoma development on an epithelium without glandular structure has led to many theories. The metaplastic theory seems to unite many authors.

Mechanical or chemical irritant factors lead to a metaplastic transformation of urothelial lining [5, 6].

Histologically tumor lesions form a glandular structure similar to colonic adenocarcinoma. Grignon proposed a classification into 6 histological types [6]: enteric ADK, mucinous ADK, kitten ring cell ADK, clear-cell ADK, mixed ADK, and undifferentiated ADK.

In our study we found a cell-ring kitten variant (1 case), mucinous adenocarcinoma (4 cases), and clear-cell ADK (1 case).

Macroscopic hematuria remains the main sign found in 90% of cases. Irritation bladder sign as pollakiuria or urinary burns can be found. Mucosuria is a very suggestive sign reported in 25% of the cases [7].

Cystoscopy shows often a trigonal or posterior wall single solid or ulcerative lesion.

TURB followed up by histological study confirms the diagnosis. An immunhistochemical analysis by markers is important to differentiate a primary bladder adenocarcinoma from a secondary adenocarcinoma. Such markers are cytokeratin CK7- CK 20- CDX2 and especially *β*-catenin.

Total cystectomy with extensive lymph node dissection remains the gold standard. Partial cystectomy is an option for mobile bladder part tumor, with often disappointing results [8, 9]. Based on results obtained with colonic adenocarcinomas, some authors propose 5-fluorouracil (5-FU) chemotherapy, with fickle results [10–12]. Neoadjuvant chemotherapy based on platinum salts can be used for locally advanced tumors.

### 4.3. Sarcomatoid Bladder Carcinoma

Rare type represents less than 0.5% of bladder tumors.

Histologically we found the coexistence of an epithelial urothelial contingent and spindle cells with sarcomatous connective appearance predominant contingent.

It is a very aggressive type, often diagnosed at an advanced stage. It mainly affects men with an average age of 66.4 years [13, 14]. Three etiological factors have been suggested: radiation, smoking, and cyclophosphamide.

Symptomatology is dominated by hematuria associated or not with irritative signs.

Positive diagnosis is based on the anatomopathological study of the resection chips associated with immunohistochemical study: positive immunostaining with epithelial markers (cytokeratins, EMA).

Radical cystectomy with pelvic lymph node dissection is widely described in most published cases [13, 14]. Perret et al. underline that patients that survived the longer (up to 12 years) were initially treated by radical cystectomy [14]. Radical cystectomy is sometimes associated with postoperative radiotherapy. This tumor type is effectively radiosensitive [13]. Metastatic disease management is based on chemotherapy.

### 4.4. Neuroendocrine Vesical Carcinoma

First described in 1981 by Cramer et al. [15, 16], it represents 0.5 to 1% of all bladder tumors and usually occurs in the elderly, between the fifth and ninth decade with a male predominance (sex ratio of 3.6) [15].

Pathogenesis hypothesis is as follows: a malignant transformation of neuroendocrine cells normally presents urothelium, a totipotent stem cell, and a urothelial metaplasia.

Tumors with a neuroendocrine contingent are classified into two groups:

- Pure NET: more than 90% of tumor cells express neuroendocrine differentiation.
- Mixed NET: the neuroendocrine contingent represents between 10 and 90% of the tumor. The presence of neuroendocrine cells dispersed within a tumor (neuroendocrine contingent <10%) has no particular prognostic value.

Smoking was reported in 67% of the cases. However, correlation between tobacco and bladder small cell carcinoma is not as important as for small cell lung carcinoma.

Macroscopic hematuria is the main sign, associated with irritative urinary signs. Some cases report paraneoplastic syndrome as Lambert Eaton syndrome, Cushing's syndrome, etc.

Anatomopathological examination shows small cells with an irregular hyperchromatic nucleus, often in mitosis, arranged in elongated spans with necrosis.

Immunohistochemistry confirms the diagnosis and reveals the expression of at least one neuroendocrine marker: NSE (+), chromogranin (+), and synaptophysin (+).

In our series, synaptophysin immunostaining was positive in all cases, associated with positive labeling with chromogranin (3 cases) or cytokeratin in the urothelial component (1 case) or CD56 (1 case).

### 4.5. The Bladder Sarcomas

Rare entities represent less than 1% of bladder cancers [17, 18]. Those are very aggressive tumors with grim prognosis. The most common types are leiomyosarcoma and rhabdomyosarcoma. There were 3 cases of leiomyosarcoma in our series.

#### 4.5.1. Leiomyosarcoma

It is a malignant mesenchymal tumor with smooth muscle differentiation. Bladder location is extremely rare and occurs in both children and adults with a maximum incidence beyond 60 years and a male preference with a sex ratio 3/1.

Etiopathogenesis remains unknown. Several factors have been described as malignant transformation of a leiomyoma into leiomyosarcoma [19], chronic bladder irritation, some chemical carcinogens, the mutation of the retinoblastoma gene, radiation [20], and hereditary factors in 1% of cases, as well as long-term chemotherapy with cyclophosphamide [21].

Leiomyosarcoma has a significant local extension potential, before metastasis.

Positive diagnosis based on anatomopathological study reveals tangled bundles of spindle cells with more or less marked nuclear atypia. Immunohistochemistry usually shows strong expression of muscle markers as smooth muscle *α*-actin and HHF35. Surprisingly the desmin is less frequently positive.

Total cystectomy preceded by neoadjuvant chemotherapy seems to be the best option. Radiotherapy can be used as adjuvant to surgery for high grade tumor (2-3), size >5 cm, or R1-R2 marging with no possibility of recovery [22].

#### 4.5.2. Rhabdomyosarcoma

Exceptional tumor with no sex ratio or age predilection: there are many reports on rhabdomyosarcomas radiation induced case especially after other cancer treatments.

Hematuria is the main symptom. Abdominal mass or dysuria can lead to the diagnosis.

The embryonic histological type is most frequent. The botryoid forms with intraluminal polypoid growth have better prognosis than invasive growth form.

Treatment is based on surgery and irradiation. Chemotherapy is here used to facilitate local treatment rather than to prevent metastases [23].

#### 4.5.3. Angiosarcomas

Bladder localization of angiosarcomas is exceptional. It occur at endothelial vessels cells expense. Those are very aggressive tumors often diagnosed at locally advanced or metastatic stage, with a male predilection. Some risk factors have been described like pelvic irradiation, exposition to vinyl chloride, and arsenic.

Anatomopathological study shows structures resembling anastomosed vessels, filled with blood and bordered by atypical endothelial cells [24].

#### 4.5.4. Other Sarcomas

Cases of osteosarcomas, malignant fibrous histiocytomas, liposarcomas, and primary neuroectodermal tumors have been described [25].

## 5. Conclusion

Bladder tumors arouse particular interest in urological carcinology because they represent a diagnosis challenge due to their frequency, pathological polymorphism, precise staging difficulty, and grim prognosis.

Rare bladder tumors include histological entities which represent for each of them less than 5% of bladder tumors. The rarity and small size of these tumors justify the absence of clear and consensual therapeutic management.

Hematuria is the main common clinical sign. Diagnosis should be done as soon as possible due to the poor prognosis, with a 5-year overall survival rate of 33.3% in our study.

Diagnosis is based on an anatomopathological bladder tissue examination obtained by TURB.

No role of total cystectomy concerning the conclusions could be drawn from this series because of the small number of patients undergoing the operation, but a review of the literature suggests this may be the treatment of choice. Our data reaffirm the highly aggressive nature of those lesions. Therefore, aggressive and fast therapy is justified when feasible and gives the best outcomes.

## Figures and Tables

**Table 1:** Presenting symptoms and physicals examinations' results.

	Number of patients	%

Total hematuria	25	93
(i) Painless hematuria	14	51.9
(ii) Hematuria and frequency/dysuria	11	40.7
Acute urinary retention	1	3.7
Hypogastric budding lesions	1	3.7

Normal physical examination	6	37
Abnormal physical examination	21	77

**Table 2:** Physical examination abnormal sign.

Abnormal physical examination sign	29	—
(i) Hypogastric mass	4	13.8
(ii) Mattery hypogastric fistula	2	6.9
(iii) Bladder globe	4	13.8
(iv) Hypogastric sensitivity	3	10.3
(v) Previous laparotomy scar	1	3.4
(vi) Groans on auscultation	3	10.3
(vii) Enlarged prostate	7	24.1
(viii) Pelvic shield	1	3.4
(ix) Anterior vaginal wall's infiltration	4	13.8

**Table 3:** Cumulative distribution of tumors at endoscopy.

Localization	Number	%
Anterior wall	4	7.8
Vault	6	11.8
Right wall	9	17.6
Left wall	12	23.5
Trigonal	14	27.5
Posterior wall	5	9.8
Bladder neck	1	2.0

**Table 4:** Tumor's grading at the diagnosis.

	Number	%
T1	3	11
T2	10	37
T3	7	26
T4	7	26
Tx	0	0

**Table 5:** Survival depending on tumor histological type at 1 and 5 years.

Histological type	Number of cases	%	1-year survival %	5-year survival %	Patient lost to follow-up
Adenocarcinoma	6	22	50.0	33.3	2
Leiomyosarcoma	3	11	66.6	33.3	0
Neuroendocrine carcinoma	5	19	20	0	1
Sarcomatoid carcinoma	3	11	66.6	33.3	1
Epidermoid carcinoma	10	37	70.0	50.0	2
Overall	27	100	55.6	33.3	6

## Data Availability

The data collected for carrying out this work come from patient files in the department's archives and operating reports.

## Abbreviations

MRI: Magnetic resonance imaging
IVU: Intravenous urography
TURB: Transurethral resection of the bladder
ADK: Adenocarcinoma
CK: Cytokeratin
CDX2: Caudal-type homeobox 2
5-FU: 5-Fluorouracil
EMA: Epithelial Membrane Antigen
NET: Neuroendocrine tumor
NSE: Neuron-specific enolase
CD 56: Cluster of differentiation 56.

## References

[1] Siegel R. L., Miller K. D., Jemal A. (2018). Cancer statistics. *Cancer Journal for Clinicians*.

[2] Uhry Z., Remontet L., Colonna M. (2013). Cancer incidence estimation at a district level without a national registry: a validation study for 24 cancer sites using French health insurance and registry data. *Cancer Epidemiology*.

[3] Rundle J. S. H., Hart A. J. L., McGeorge A., Smith J. S., Malcolm A. J., Smith P. M. (1982). Squamous cell carcinoma of bladder. A review of 114 patients. *British Journal of Urology*.

[4] Bejany D. E., Lockhart J. L., Rhamy R. K. (1987). Malignant vesical tumors following spinal cord injury. *Journal of Urology*.

[5] Debbagh A., Bennani S., Hafiani M., el Mrini M., Benjelloun S. (2000). Primary adenocarcinoma of the bladder: report of a case. *Annales d’Urologie*.

[6] Grignon D. J., Ro J. Y., Ayala A. G., Johnson D. E. (1991). « Primary adenocarcinoma of the urinary bladder: a clinicopathologic analysis of 72 cases. *Cancer*.

[7] Zerbib M., Bouchot O. (2002). Adenocarcinoma of the bladder. *Progrès en Urologie*.

[8] Thomas D. G., Ward A. M. (1971). A study of 52 cases of adenocarcinoma of the bladder. *BJU International*.

[9] Bennett J. K., Wheatley J. K., Walton K. N. (1984). 10-year experience with adenocarcinoma of the bladder. *The Journal of Urology*.

[10] Nevin J. E., Melnick I., Baggerly J. T., Easley C. A., Landes R. (1974). Advanced carcinoma of bladder: treatment using hypogastric artery infusion with 5-fluorouracil, either as a single agent or in combination with bleomycin or adriamycin and supervoltage radiation. *The Journal of Urology*.

[11] Logothetis C. J., Samuels M. L., Ogden S. (1985). Chemotherapy for adenocarcinomas of bladder and urachal origin: 5-Fluorouracil, doxorubicin, and mitomycin-C. *Urology*.

[12] Hatch T. R., Fuchs E. F. (1989). Intra-arterial infusion of 5-fluorouracil for recurrent adenocarcinoma of bladder. *Urology*.

[13] Lopez-Beltran A., Pacelli A., Rothenberg H. J. (1998). Carcinosarcoma and sarcomatoid carcinoma of the bladder: clinicopathological study of 41 cases. *Journal of Urology*.

[14] Perret L., Chaubert P., Hessler D., Guillou L. (1998). Primary heterologous carcinosarcoma (Metaplastic carcinoma) of the urinary bladder. *Cancer*.

[15] Ghadouane M., Zannoud M., Alami M. (2003). Tumeur neuro-endocrine à petites cellules de la vessie. Une nouvelle observation. *Annales d’Urologie*.

[16] Navarra S., Pfister C., Gobet F., Cappele O., Planet M., Grise P. (1999). Tumeur neuroendocrine maligne de vessie: une entité à ne pas méconnaître. *Progrès en Urologie*.

[17] Dahm P., Gschwend J. E. (2003). Malignant non-urothelial neoplasms of the urinary bladder: a review. *European Urology*.

[18] Manunta A., Vincendeau S., Kiriakou G., Lobel B., Guillé F. (2005). Non-transitional cell bladder carcinomas. *BJU International*.

[19] Stigssen B. M. (1999). Leiomyosarcoma of the bladder in a patient with von Recklinghausen disease. *Ugeskr Laeger*.

[20] Berdjis C. C. (1971). *Pathology of Irradiation*.

[21] Trasher J. B. (1990). Bladder leiomyosarcoma following cyclophosphamide therapy for lupus nephritis. *Journal of Urology*.

[22] Group EESNW (2014). Soft tissue and visceral sarcomas: ESMO Clinical Practice Guidelines for diagnosis, treatment and follow-up. *Annals of Oncology*.

[23] Atmani Y. (2017). les tumeurs de vessie chez l’enfant à propos de 05 cas.

[24] Boyle H., Fléchon A., Droz J.-P. (2010). Tumeurs rares de la vessie. *Bulletin du Cancer*.

[25] Lott S., Lopez-Beltran A., Montironi R., MacLennan G. T., Cheng L. (2007). Soft tissue tumors of the urinary bladder. *Human Pathology*.

